# Analysis of genetic population structure and diversity in *Mallotus oblongifolius* using ISSR and SRAP markers

**DOI:** 10.7717/peerj.7173

**Published:** 2019-06-21

**Authors:** Wuping Yan, Juanling Li, Daojun Zheng, Cynthia Friedman, Huafeng Wang

**Affiliations:** 1 Institute of Tropical Agriculture and Forestry, Hainan University, Haikou, China; 2 Institute of Tropical Horticulture, Hainan Academy of Agricultural Sciences, Haikou, China; 3 The Department of Biological and Environmental Sciences, Concordia University of Edmonton, Edmonton, Canada

**Keywords:** SRAP, *Mallotus oblongifolius*, ISSR, Genetic diversity, Genetic structure

## Abstract

**Background:**

*Mallotus oblongifolius*, an evergreen shrub endemic to Hainan Island, China, is important both medicinally and economically. Due to its special medicinal significance and the continuing rise of market demand, its populations in the wild have been subject to long-term illegal and unrestrained collection. Hence, an evaluation of genetic variability is essential for the conservation and genetic reserve development of this species.

**Methods:**

Sequence-related amplified polymorphism (SRAP) and inter-simple sequence repeat (ISSR) markers were employed to assess the genetic diversity and genetic structure of 20 natural populations of *M. oblongifolius* growing in different eco-geographical regions of Hainan Island, China.

**Results:**

We revealed a considerable genetic diversity (*h* = 0.336, *I* = 0.5057, SRAP markers; *h* = 0.3068, *I* = 0.4657, ISSR markers) and weak genetic differentiation (Gst = 0.2764 for SRAP, Gst = 0.2709 for ISSR) with the same gene flow (Nm = 1.3092 for SRAP, Nm = 1.346 for ISSR) among the *M. oblongifolius* populations. The Mantel Test showed that the distribution of genetic variation among populations could not be explained by the pronounced geographical distances (*r* = 0.01255, *p* = 0.5538). All results of the Unweighted Pair Group Method with Arithmetic Mean (UPGMA), Neighbor-joining (NJ), Principal Coordinate Analysis (PCoA) and Bayesian analyses supported a habitat-specific genetic clustering model for *M. oblongifolius*, indicating a local adaptive divergence for the studied populations.

**Discussion:**

We suggested that the habitat fragmentation and specificity for *M. oblongifolius* populations weakened the natural gene flow and promoted an adaptation to special habitats, which was the main reason for local adaptive divergence among *M. oblongifolius*.

## Introduction

*Mallotus oblongifolius* is a tropical plant belonging to Euphorbiaceae. This plant, native to regions of Hainan Island in China, is economically important and medicinally relevant. *Mallotus oblongifolius* is an evergreen shrub or small tree (2–10 m), mostly dioecious and rarely monoecious (Lin & Zhou, 1992). Its leaves, which are commonly known as “Zhe Gu Cha,” have been used in a popular herbal tea to promote digestion. They are also used as a folk medicine for the treatment of cholecystitis. Various other biological properties of *M. oblongifolius* leaf extracts have been recently reported, and extracts have been shown to have anti-atherosclerotic (Yueli et al., 2011), analgesic and antioxidant properties. Due to its special medicinal significance, *M. oblongifolius* is known as “glossy ganoderma” in China. During the field investigations of this species, we observed considerable morphological variation. Such variation included leaf color, ranging from purple-red, red/pale red to pale green/green/dark green; size and width of leaves. Thus, to ensure that this plant is being used sustainably in a way that preserves phenotypic diversity, its germplasm must be collected, conserved and assessed at the phenotypic, genetic and molecular levels.

In previous studies of *M. oblongifolius*, researchers mainly focused on effective chemical composition analysis (Wei et al., 2004), product processing and pharmacology values. As a result, little information exists about *M. oblongifolius* diversity, germplasms and breeding. Furthermore, due to its special medicinal significance, wild populations have long been subjected to illegal and unrestrained collection as a result of the continuing rise of market demand, and the number of *M. oblongifolius* individuals has dropped dramatically. In our previous field investigations, healthy populations were hard to find. Additionally, some *M. oblongifolius* habitats have been exploited as tourist areas or have been reclaimed for agriculture, resulting in the disappearance of wild populations. Thus, the abundance and genetic variability of this important species may soon vanish if proper action is not taken. An evaluation of genetic variability is essential for the conservation and development of genetic reserves for this species. To ensure their success, these conservation strategies (in situ and ex situ) must be based on scientific practices (Bruni et al., 2013; Gentili et al., 2015). The analysis done here represents a preliminary step for selecting breeding material and establishing conservation strategies.

A molecular marker is a genetic marker based on nucleotide sequence variation in genetic material between individuals, and is a direct reflection of genetic polymorphism at the DNA level. It is a powerful tool for estimating the characteristics of genetic diversity and distinguishing among individuals from different sources (EL-Bakatoushi & Ahmed, 2018; Nair et al., 2018; Kumar & Agrawal, 2017; Zheng et al., 2012). Sequence-related amplified polymorphism (SRAP) and inter-simple sequence repeats (ISSRs) markers are dominant markers based on PCR and have been widely used in different genetic diversity studies (Xing et al., 2016; Gaafara et al., 2017; Sun et al., 2018; Khalifa et al., 2016). Sequence-related amplified polymorphism is a PCR-based molecular marker developed by Li & Quiros (2001), which uses a unique dual primer design to amplify specific regions of open reading frames (ORFs). It has been used in genetic diversity analysis and genetic map construction studies for a wide range of plants. Inter-simple sequence repeat is a similarly powerful tool for genetic mapping, gene tagging, germplasm resource identification, genetic distance, genetic diversity and molecular marker-assisted breeding research. Inter-simple sequence repeat markers consist of repeating units of one to six base pairs, targeting regions between two simple sequence repeat (SSR) sequences (Zietkiewicz, Rafalski & Labuda, 1994).

In this study, we used SRAP and ISSR markers to assess the genetic diversity and genetic structure of 20 natural populations of *M. oblongifolius* on Hainan Island, China. The targets of this study were: (i) to assess the level of genetic diversity and genetic differentiation across *M. oblongifolius* populations; (ii) to characterize the genetic structure within the populations and (iii) to assess the correlation between the two markers. These results could benefit *M. oblongifolius* germplasm collection, conservation and future breeding.

## Materials and Methods

### Plant materials and DNA extraction

**Figure 1:**
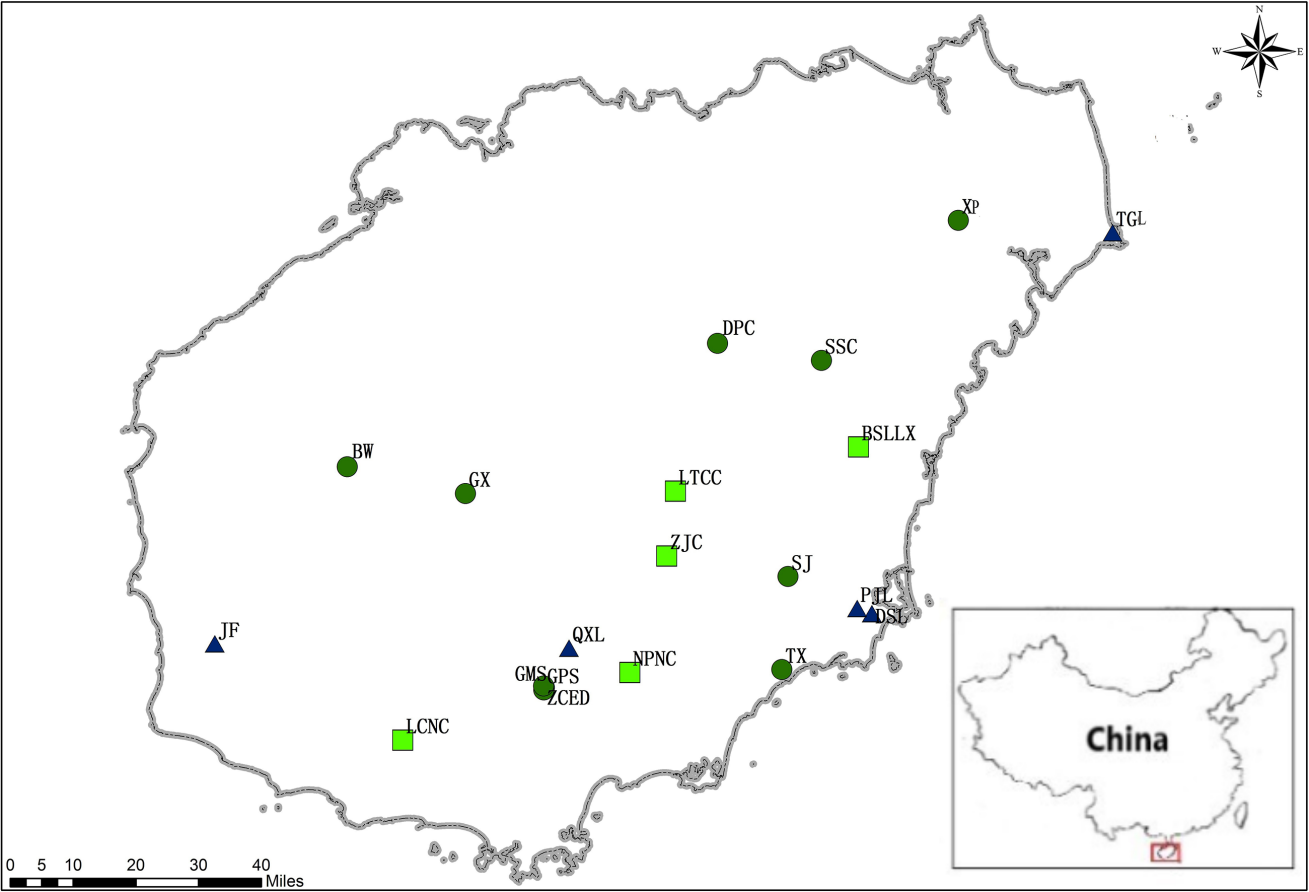
Locations of *M. oblongifolius* populations sampled in the current study (see Table 1 for explanation of population abbreviations). Sampled populations from different habitats are marked with different colors: populations along stream banks (dark-green circles), populations along the mountainside (blue triangle), populations found at the foot of the mountain (green square).

In total, 371 adult individuals of *M. oblongifolius* were collected from 20 naturally occurring populations on Hainan Island, China from January 2016 to May 2017 (Fig. 1; Table 1), covering almost the entire range of species distribution on Hainan Island. For the convenience of the study, their names have been assigned arbitrarily, with populations BW, DPC, GMS, GPS, GX, SJ, SSC, TX, XP and ZCED along stream banks; populations DSL, JF, PJL, QXL and TGL along the mountainside and populations BSLLX, LCNC, LTCC, NPNC and ZJC at the foot of the mountain. The longitude and latitude of each population were recorded using a global positioning system (GPS; UniStrong, Beijing, China). Eight to 22 individuals from each of the 20 populations were used for SRAP and ISSR analyses. Individuals were randomly sampled from known geographic areas of each population and were spaced at least five meters apart to minimize sampling of progeny.

**Table 1:** Location, population abbreviation, coordinates, number of sampled plants per population (N) and habitat type of the 20 studied populations of *M. oblongifolius* in Hainan Island, China.

Population	Location	Latitude	Longitude	Habitat	*N*
BSLLX	Baishi mountain, Qionghai	19°10′18.13″	110°23′36.17″	At the foot of the mountain	20
BW	Bawang mountain, Changjiang	19°07′3.81″	109°08′52.64″	Along stream banks	14
DPC	Dapo mountain, Tunchang	N19°24′36.27″	E110°02′53.74″	Along stream banks	20
DSL	Dongshan mountain,Wanning	N18°47′8″	E110°25′38″	Along mountainside	20
GMS	Guomi mountain, Baoting	N18°36′38.72″	E109°37′56.94″	Along stream banks	19
GPS	Gupi mountain, Baoting	N18°36′18.16″	E109°37′52.60″	Along stream banks	20
GX	Gexin village, Baisha	N19°03′ 30.51″	E109°26′11.92″	Along stream banks	19
JF	Jianfeng mountain, Ledong	18°42′14.99″	108°49′50.23″	Along mountainside	20
LCNC	Lichai farm, Sanya	N18°29′ 11″	E109°17′23″	At the foot of the mountain	20
LTCC	Lingtou tea plantation, Qiongzhong	N19°04′2.55″	E109°56′51.61″	At the foot of the mountain	20
NPNC	Nanping farm, Lingshui	N18°38′ 49″	E109°50′23″	At the foot of the mountain	16
PJL	Pengjia mountain,Wanning	N18°47′50″	E110°23′29″	Along mountainside	8
QXL	Qixian mountain, Baoting	N18°42′7″	E109°41′30″	Along mountainside	15
SJ	Sanjiaoshui farm,Wanning	N18°52′16″	E110°13′23″	Along stream banks	20
SSC	Shenshui village, Dingan	N19°22′ 18″	E110°18′9″	Along stream banks	20
TGL	Tonggu mountain, Wenchang	19°40′5.72″	111°00′45.57″	Along mountainside	18
TX	Tianxin village, Wanning	N18°39′20″	E110°12′33″	Along stream banks	21
XP	Xinpo town, Haikou	N19°53′28″	E111°8′35″	Along stream banks	20
ZCED	Zhacun village, Baoting	N18°36′52.18″	E109°37′50.57″	Along stream banks	22
ZJC	Zhajie village, Qiongzhong	N18°55′02.28″	E109°55′39.05″	At the foot of the mountain	19

A modified cetyltrimethylammonium bromide method (Li et al., 2010) was used to extract genomic DNA from fresh and healthy leaves of each *M. oblongifolius* population. The value of absorbance of the extracted DNA was measured using an ultraviolet spectrophotometer, and its integrity was detected with agarose gel electrophoresis. DNA samples were diluted to a final concentration of 20 ng/μL for PCR analysis.

### SRAP-PCR analysis

Among 100 pairs of SRAP primer combinations, 14 pairs with good repeatability and high polymorphism were selected to amplify the genomic DNA of the 371 accessions from the 20 naturally occurring populations of *M. oblongifolius* by SRAP-PCR (Table S1). According to Yan et al. (2018), the optimum SRAP-PCR reaction system (20 μL) includes 2.0 μL 10 × PCR buffer, 3 U Taq DNA polymerase, 150 μmol/L dNTPs, 0.4 μmol/L each of SRAP forward and reverse primers and 40 ng genomic DNA. PCR reactions were carried out in a Heal Force T960 Thermocycler (Biometra, Shanghai, China) under the following conditions: initial denaturation at 94 °C for 5 min followed by five cycles of denaturation at 94 °C for 1 min, annealing at 35 °C for 1 min and extension at 72 °C for 1 min. For the next 35 cycles, denaturation at 94 °C for 1 min, annealing at 48 °C for 1 min and extension at 72 °C for 1 min; a final extension step at 72 °C for 10 min. The amplification products were resolved on 2% agarose gels.

### ISSR-PCR analysis

Among 100 ISSR primers, 10 primers with good repeatability and high polymorphism were selected to amplify the genomic DNA of the 371 accessions from the 20 naturally occurring populations of *M. oblongifolius* by ISSR-PCR (Table S2). The optimum ISSR-PCR reaction system (20 μL) includes 2.0 μL 10 × PCR buffer, 1.5 U Taq DNA polymerase, 300 μmol/L dNTPs, 0.6 μmol/L ISSR primers and 80 ng genomic DNA. A Heal Force T960 Thermocycler (Biometra, Shanghai, China) was used for ISSR-PCR. PCR cycling conditions were: initial denaturation at 94 °C for 4 min followed by 40 cycles of denaturation at 94 °C for 40 s, annealing at 50 °C for 45 s (for annealing at primer-specific temperatures, see Table S2) and extension at 72 °C for 2 min; a final extension step at 72 °C for 8 min. The amplification products were resolved on 2% agarose gels.

### Data analysis

Bands were scored manually. According to the weight of the DNA ladder (100 bp), the same weight bands were marked as a line. The bands that were clearly visible and repeatable on the electrophoresis map were marked as “1” and the absence of a band at the same site was marked as “0”. A binary data matrix was compiled for each primer set. Both the total number of bands amplified by each primer and the number of polymorphic bands were calculated. Most of the following analyses were carried out based on adjoined matrix data.

The genetic diversity of different *M. oblongifolius* populations was evaluated by calculating the number of alleles (na), effective number of alleles (ne), Nei’s gene diversity (h), Shannon’s information index (I) and percentage of polymorphism bands (PPB). These parameters (na, ne, h, I, PPB) were calculated using POPGENE (version 1.32) software (Yeh et al., 1999). The total genetic diversity (Ht), the mean within-population genetic diversity (Hs), genetic differentiation coefficients among different populations (Gst) (Nei, 1974) and gene flow number (Nm, Nm = 0.5(1 − Gst)/Gst) were calculated according to the gene frequency matrix using POPGENE (version 1.32) software. Genetic similarity (GS) coefficients and genetic distance (GD) among the 20 *M. oblongifolius* populations were calculated using Jaccard’s similarity coefficients (Sneath & Sokal, 1973). The Unweighted Pair Group Method with Arithmetic Mean (UPGMA) and Neighbor-joining (NJ) trees based on the GD were used for cluster analysis on Molecular Evolutionary Genetics Analysis (MEGA) version 6.06 (Tamura et al., 2013). Principal Coordinate Analysis (PCoA) based on the GS matrix was performed using NTSYS-pc version 2.1 (Rohlf, 2000) to detect the genetic relationships among populations. The MXComp program of NTSYS-pc version 2.1 was used to test ISSR and SRAP data by Mantel (Mantel, 1967) statistics. Correlations between the two GD matrices were compared and a significance test was conducted. A Mantel test was performed to estimate a correlation between the matrices of Nei’s (1978) GDs and of geographical distances using NTSYS-pc version 2.1. Geographic distances among populations were measured based on the latitude and longitude coordinates of the populations.

The population structure of *M. oblongifolius* was studied further. A calculation based on a Bayesian model was performed on the combined SRAP and ISSR data in STRUCTURE program version 2.3.4 (Pritchard, Stephens & Donnelly, 2000) to detect the number of population clusters (*K*). The most likely number of clusters (*K*) was estimated using STRUCTURE HARVESTER (Earl & Vonholdt, 2012), which determines the optimal *K* depending on the probability of the data for a given *K* and the Δ*K* (Evanno, Regnaut & Goudet, 2005).

## Results

### Polymorphism analysis of SRAP and ISSR amplified products

**Figure 2:**
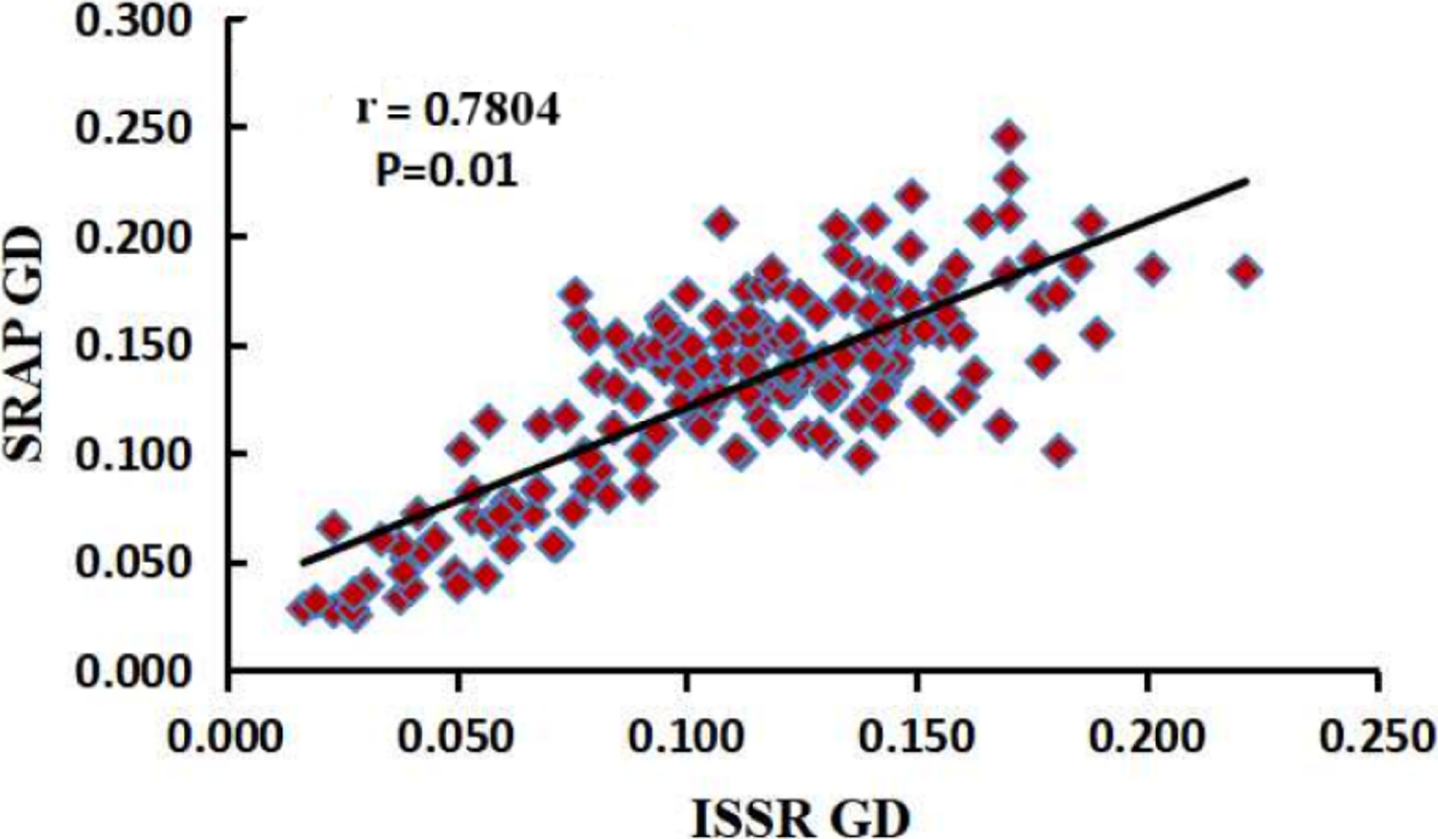
Correlation analysis between the two genetic distances matrices of SRAP and ISSR.

The number of plants sampled per population of *M. oblongifolius* is shown in Table 1. Fourteen SRAP primers and 10 ISSR primers were selected to study genetic diversity and genetic differentiation of *M. oblongifolius*. One hundred and ninety-seven total bands were produced by 14 SRAP primers, of which 164 bands (83.24%) were polymorphic (see Table S1). Nine to 20 bands and three to 19 polymorphic bands were yielded for each primer pair. The average number of bands and polymorphic bands for each primer pair was 14.1 and 11.7, respectively. For the ISSR markers, the 10 primers generated 141 bands, and each primer yielded 10–17 bands (see Table S2). There was an average of 14.1 bands per primer set, and 12.4 polymorphic loci were obtained (87.94%). These results show that both SRAP and ISSR markers can effectively reveal the polymorphism among materials. Mantel’s test showed that at the population level, the analysis results of SRAP and ISSR were highly and significantly correlated (*r* = 0.7804, *p* = 0.01) (Fig. 2). Thus, SRAP and ISSR are highly consistent and reliable tools for analysis of both the genetic diversity and the population structure of *M. obongifolius*. However, ISSR exceeded SRAP in the ability to detect genetic polymorphism with higher resolving power. The PPB ranged from 76.47% to 100%, with an average of 87.94%, for ISSR, while SRAP ranged from 33.33% to 100%, with an average of 83.24%.

### Genetic diversity and genetic differentiation

POPGENE 1.32 was used to analyze genetic diversity and genetic differentiation from both SRAP and ISSR data (Tables 2 and 3). For the SRAP analysis, the effective number of alleles (ne), Nei’s gene diversity (h) and Shannon’s information index (I) at the species level were calculated to be 1.5702, 0.336 and 0.5057, respectively. Assuming a Hardy–Weinberg equilibrium, the effective number of alleles per locus (ne) ranged from 1.3415 to 1.4865, with an average of 1.42155; Nei’s gene diversity (h) ranged from 0.1957 to 0.2796, with an average of 0.243635; Shannon’s information index (I) ranged from 0.2855 to 0.4117, with an average of 0.36174 and the PPB ranged from 49.24% to 79.19%, with an average of 67.31%. For the ISSR analysis, Nei’s gene diversity (h) ranged from 0.1529 to 0.2903, with an average of 0.222785; Shannon’s information index (I) ranged from 0.2236 to 0.4303, with an average of 0.33383; the PPB ranged from 39.01% to 80.85%, with an average of 65.14%. The number of alleles (Na) ranged from 1.3901 to 1.8085, with an average of 1.65142 and the effective number of alleles per locus (ne) ranged from 1.2714 to 1.5012, with an average of 1.38099. At the species level, na, ne, h, I and PPB were calculated to be 2%, 1.5186%, 0.3068%, 0.4657% and 87.94%, respectively. The ISSR markers revealed the highest genetic diversity in population XP (*p* = 80.85%, *h* = 0.2722, *I* = 0.4087), followed by populations TX and SJ, and the lowest genetic diversity was found in population PJL. According to the SRAP markers, population TX had the highest genetic diversity (*p* = 79.19%, *h* = 0.2725, *I* = 0.4074), followed by populations ZJC and SJ, and the lowest genetic diversity was found in population PJL. The genetic diversity demonstrated by the SRAP marker was similar to that revealed by the ISSR marker. At the species level, h, I and P, meaning the genetic diversity, were significantly higher than at the population level. Each index exhibited a small change among populations, and there is a similar level of genetic diversity within each population.

**Table 2:** Genetic diversity of *M. oblongifolius* populations and species based on SRAP.

Population	na	ne	h	I	PPB (%)
GPS	1.6041	1.3884	0.2225	0.3288	60.41
XP	1.7208	1.4826	0.2751	0.4044	72.08
DPC	1.6193	1.3943	0.23	0.3408	61.93
SJ	1.7259	1.4473	0.262	0.3904	72.59
TX	1.7919	1.4697	0.2725	0.4074	79.19
ZCED	1.7005	1.4175	0.2449	0.3667	70.05
GMS	1.7157	1.4409	0.2547	0.379	71.57
SSC	1.7157	1.4357	0.2539	0.379	71.57
GX	1.6345	1.3768	0.219	0.3276	63.45
BW	1.6497	1.4184	0.2409	0.3564	64.97
ZJC	1.731	1.4865	0.2796	0.4117	73.10
LCNC	1.7157	1.4309	0.2516	0.3761	71.57
BSLLX	1.6954	1.4114	0.2424	0.3633	69.54
LTCC	1.6396	1.3714	0.2156	0.3236	63.96
NPNC	1.6802	1.4613	0.26	0.3814	68.02
TGL	1.5736	1.3415	0.1983	0.296	57.36
JF	1.7107	1.4718	0.2677	0.3937	71.07
DSL	1.7056	1.4361	0.2523	0.3753	70.56
QXL	1.6396	1.3999	0.234	0.3477	63.96
PJL	1.4924	1.3486	0.1957	0.2855	49.24
Mean	1.673095	1.42155	0.243635	0.36174	67.31
Species lever	2	1.5702	0.336	0.5057	83.24

**Note:**

na: The number of alleles; ne: Effective number of alleles; h: Nei’s gene diversity; I: Shannon’s information index; PPB, Percentage polymorphism bands.

**Table 3:** Genetic diversity of *M. oblongifolius* populations and species based on ISSR.

Population	na	ne	h	I	PPB (%)
GPS	1.7447	1.441	0.258	0.3863	74.47
XP	1.8085	1.4638	0.2722	0.4087	80.85
DPC	1.6879	1.4136	0.2407	0.3592	68.79
SJ	1.7872	1.5012	0.2903	0.4303	78.72
TX	1.8014	1.4648	0.267	0.3993	80.14
ZCED	1.6525	1.3261	0.1975	0.3032	65.25
GMS	1.7376	1.3836	0.2263	0.3438	73.76
SSC	1.6667	1.3579	0.2123	0.3213	66.67
GX	1.6383	1.3539	0.2098	0.3171	63.83
BW	1.6525	1.3716	0.2201	0.3317	65.25
ZJC	1.6312	1.3554	0.2083	0.3134	63.12
LCNC	1.6241	1.3811	0.2208	0.3296	62.41
BSLLX	1.6383	1.375	0.2194	0.3288	63.83
LTCC	1.6241	1.3664	0.2143	0.3212	62.41
NPNC	1.5603	1.3293	0.193	0.289	56.03
TGL	1.5532	1.3314	0.1952	0.292	55.32
JF	1.6241	1.4051	0.2338	0.3455	62.41
DSL	1.6596	1.3852	0.2254	0.3382	65.96
QXL	1.5461	1.342	0.1984	0.2944	54.61
PJL	1.3901	1.2714	0.1529	0.2236	39.01
Mean	1.65142	1.38099	0.222785	0.33383	65.14
Species lever	2	1.5186	0.3068	0.4657	87.94

**Note:**

na: The number of alleles; ne: Effective number of alleles; h: Nei’s gene diversity; I: Shannon’s information index; PPB, Percentage polymorphism bands.

Based on SRAP and ISSR data, the total genetic diversity (Ht) and mean within-population genetic diversity (Hs) of *M. oblongifolius* were calculated, as well as the genetic differentiation coefficients among different populations (Gst) (see Table 4). Nearly 27% of the total genetic variation in the 20 populations of *M. oblongifolius* occurred among populations (Gst = 0.2764 for SRAP, Gst = 0.2709 for ISSR), rather than within. Both SRAP and ISSR markers indicated that most genetic variation in *M. obongifolius* occurs within populations. The gene flow number (Nm) of the populations of *M. oblongifolius* was 1.346 (ISSR) and 1.3092 (SRAP), which indicates that the gene exchange was high between populations. Such gene flow could reduce genetic differentiation among populations that would otherwise be caused by genetic drift.

**Table 4:** Genetic differentiation coefficients of *M. oblongifolius* populations based on ISSR and SRAP.

Marker	Ht	Hs	Gst	Nm
SRAP	0.3367	0.2436	0.2764	1.3092
ISSR	0.3056	0.2228	0.2709	1.346

**Note:**

Ht: The total genetic diversity; Hs: The mean within-population genetic diversity; Gst: The genetic differentiation coefficients among different populations; Nm: Gene flow number, Nm = 0.5(1 − Gst)/Gst.

### Cluster and population structure analysis

Both the GD and the GS among populations were calculated with POPGENE (version 1.32) using SRAP and ISSR data (see Tables S3 and S4). Based on the results of SRAP, the GD of the 20 populations of *M. oblongifolius* varied from 0.0252 to 0.2453, with an average of 0.1175. The largest GD was found in populations PJL and DPC and the smallest in populations DPC and XP, which were 0.0252 and 0.2453, respectively. Based on the results of ISSR, the GD of the 20 populations of *M. oblongifolius* varied from 0.0169 to 0.2213, with an average of 0.8911. The largest GD was found in populations PJL and ZCED, and the smallest in populations GX and SSC, which were 0.0.2213 and 0.0169, respectively. Genetic relationships among the populations were constructed by UPGMA cluster analysis based on a GD matrix using the SRAP and ISSR markers. The results show that the two markers completely divide the 20 populations of *M. oblongifolius* and that the clustering results had certain similarities but were not identical. The clustering of *M. oblongifolius* is not significantly related to its geographical origin. For example, the population GMS and population GPS with the closest geographic sources are clustered in large branches, but they are not clustered together in small branches. However, the dendrogram shows a spatial pattern of GD among the populations aligning with their habitat status.

**Figure 3:**
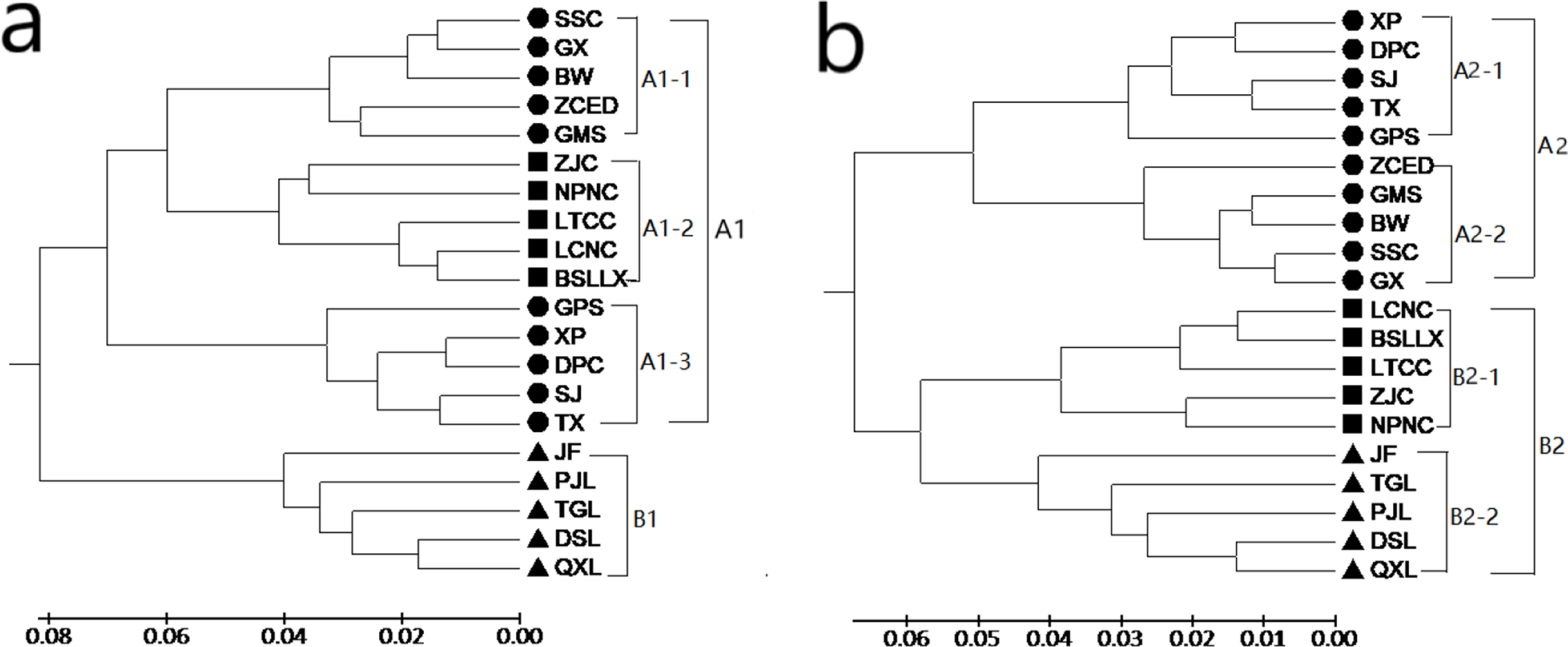
UPGMA cluster for 20 populations of *M. oblongifolius* based on SRAP data (A) and ISSR (B).

A dendrogram (Fig. 3A) that grouped the 20 populations into two main groups (Group A1 and Group B1) using the SRAP data. Group A1 consisted of populations BSLLX, BW, DPC, GMS, GPS, GX, LCNC, LTCC, NPNC, SJ, SSC, TX, XP, ZCED and ZJC, which were distributed along stream banks and the dark damp regions at the mountain bases. Three sub-groups (A1-1, A1-2 and A1-3) were identified within Group A1. Populations DSL, JF, PJL, QXL and TGL were distributed along the mountainside and tightly grouped into Group B1. The cluster analysis based on the ISSR data generated a unique dendrogram that divided the 20 populations into two main groups (Group A2 and Group B2) (Fig. 3B). Group A2 consisted of populations BW, DPC, GMS, GPS, GX, SJ, SSC, TX, XP and ZCED, whereas the other populations fell into Group B2. Two sub-groups (A2-1 and A2-2) were identified within Group A2 and two sub-groups (B2-1 and B2-2) were identified within Group B2. This was similar to the results using the SRAP markers, except for populations BSLLX, LCNC, LTCC, NPNC and ZJC. Figure 3A shows that populations BSLLX, LCNC, LTCC, NPNC and ZJC were grouped into Group A1 under SRAP results, but then were grouped into Group B2 using ISSR (Fig. 3B). This result reveals that the habitat of populations BSLLX, LCNC, LTCC, NPNC and ZJC existed between stream banks and mountainsides, thus belonging to an intermediate habitat.

**Figure 4:**
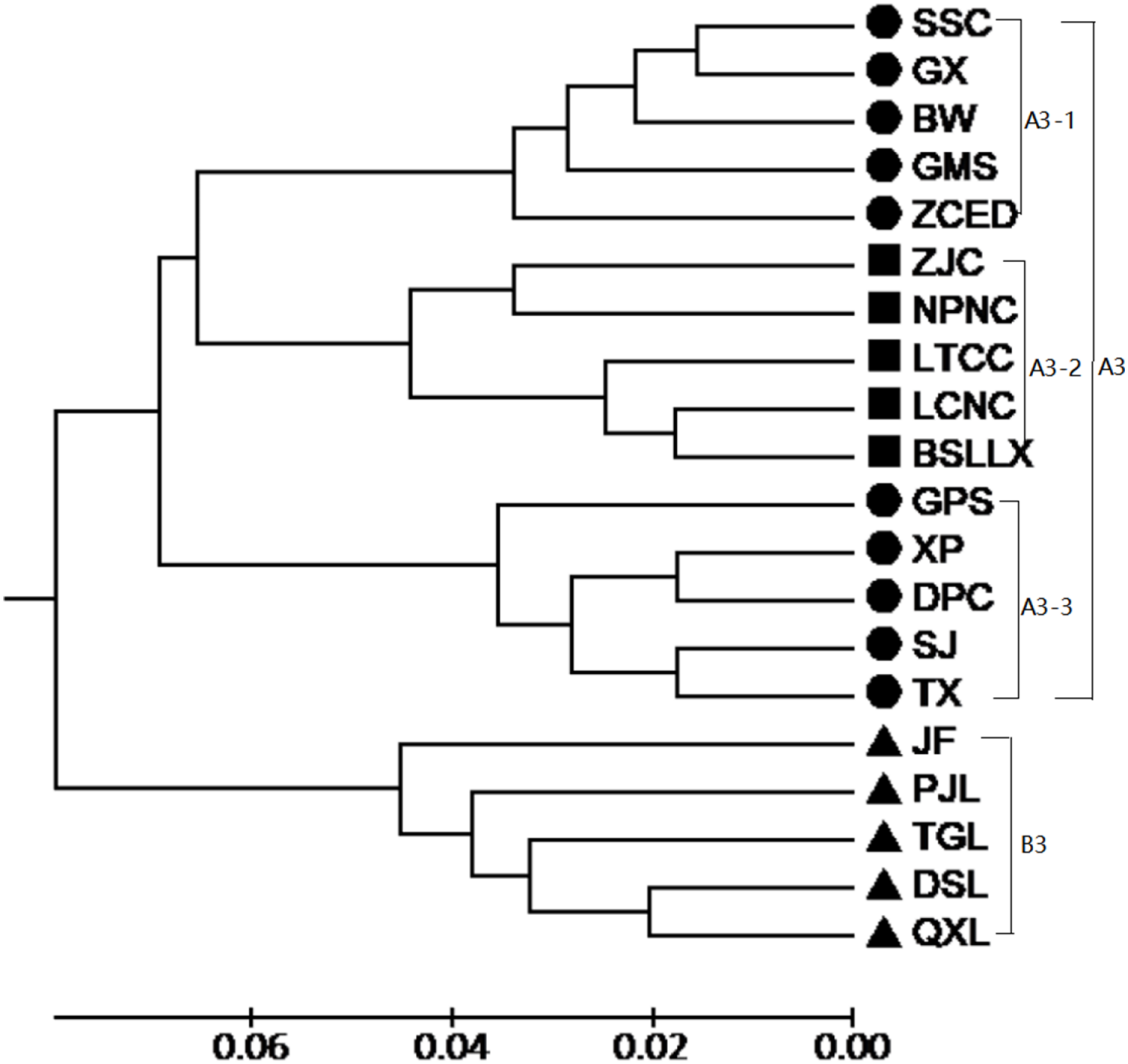
UPGMA cluster for 20 populations of *M. oblongifolius* combined SRAP and ISSR data.

**Figure 5:**
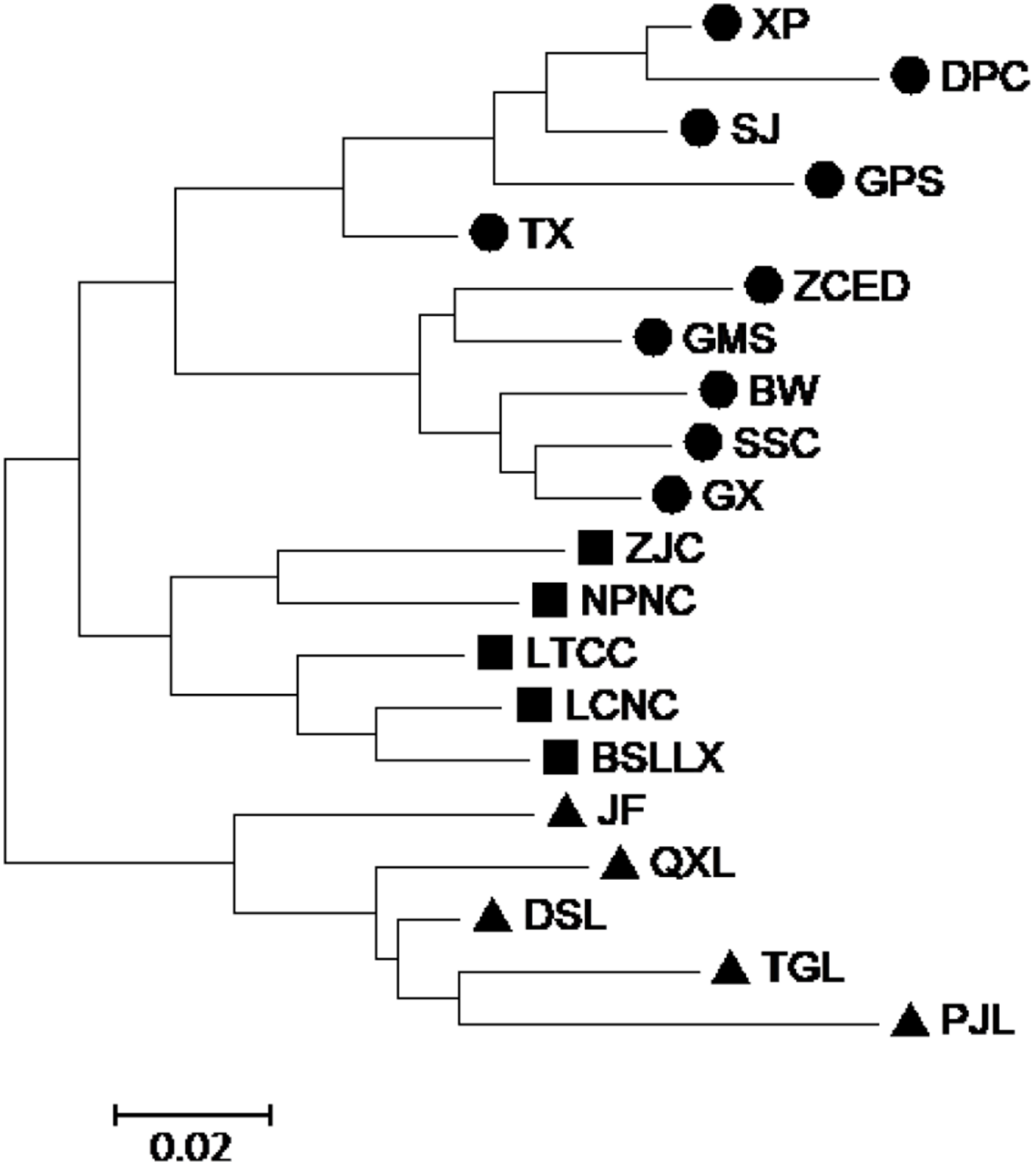
Neighbor-joining (NJ) cluster for 20 populations of *M. oblongifolius* combined SRAP and ISSR data.

According to the Mantel test, the analysis results of SRAP and ISSR were significantly correlated. Therefore, POPGENE (version 1.32) was used to calculate the GD and the GS among populations using combined SRAP and ISSR data (see Table S5). The relationships among the 20 populations of *M. oblongifolius* were revealed by UPGMA and NJ-based GD using combined SRAP and ISSR data (Figs. 4 and 5). Both UPGMA and NJ trees divided the 20 populations of *M. oblongifolius* into two major groups. The dendrogram consistent with that was constructed using the SRAP data.

**Figure 6:**
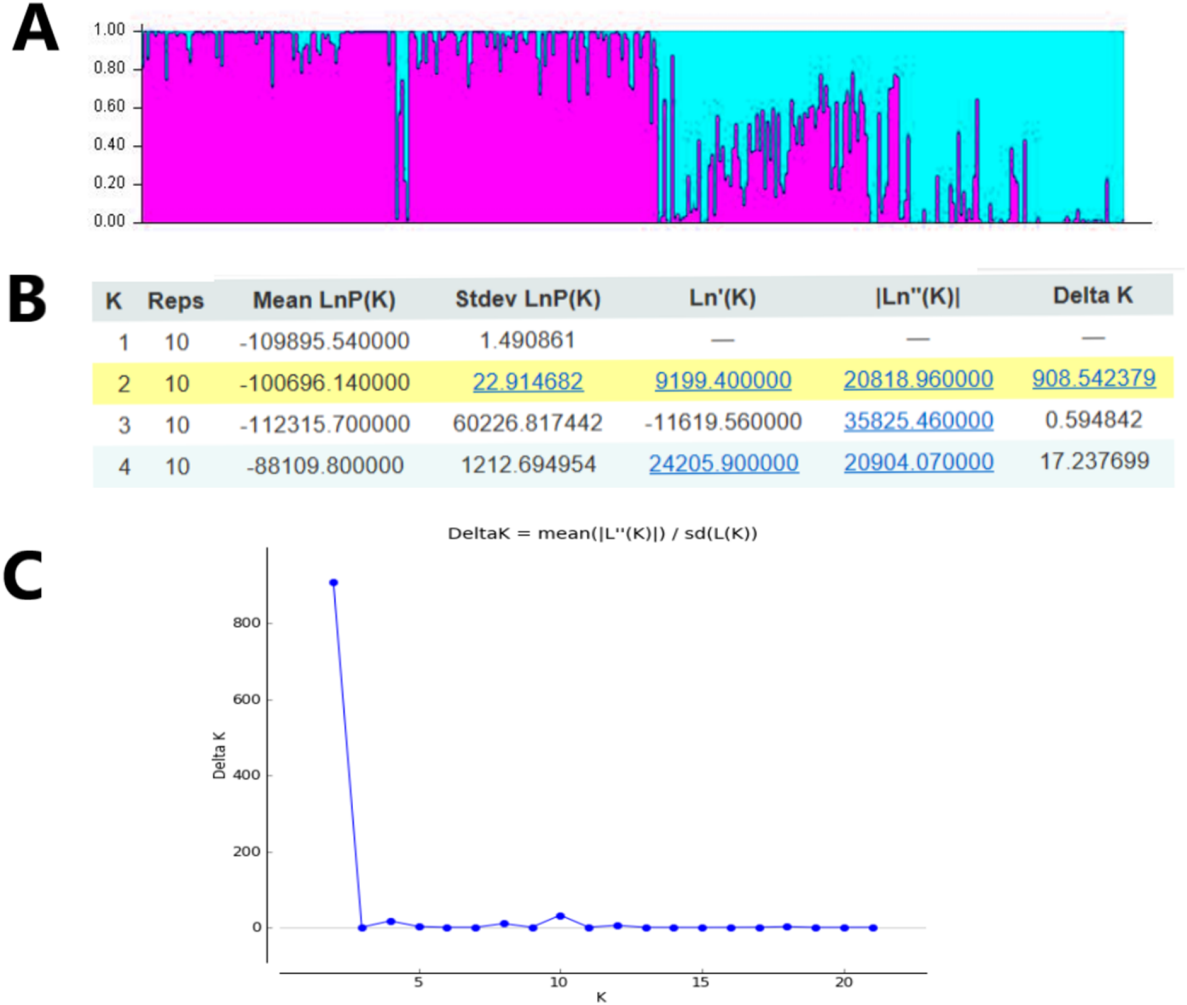
Clustering results of 20 *M. oblongifolius* populations using STRUCTURE combined SRAP and ISSR data. (A) Bar plot representation for *K* = 2, (B) Evanno table output, (C) Δ*K* values for different numbers of populations assumed (*K*) in the STRUCTURE analysis.

In order to study the population structure of *M. oblongifolius* further, we performed a calculation based on a Bayesian model on the combined SRAP and ISSR data in STRUCTURE program version 2.3.4. Results showed a spatial pattern of GDs among the populations in accordance with their habitat status, which was similar to the result of UPGMA and NJ analyses. The results showed that when *K* = 2 (Fig. 6), all the individuals of the population could be distributed to corresponding groups in a high proportion, and these groups were divided according to habitat type.

**Figure 7:**
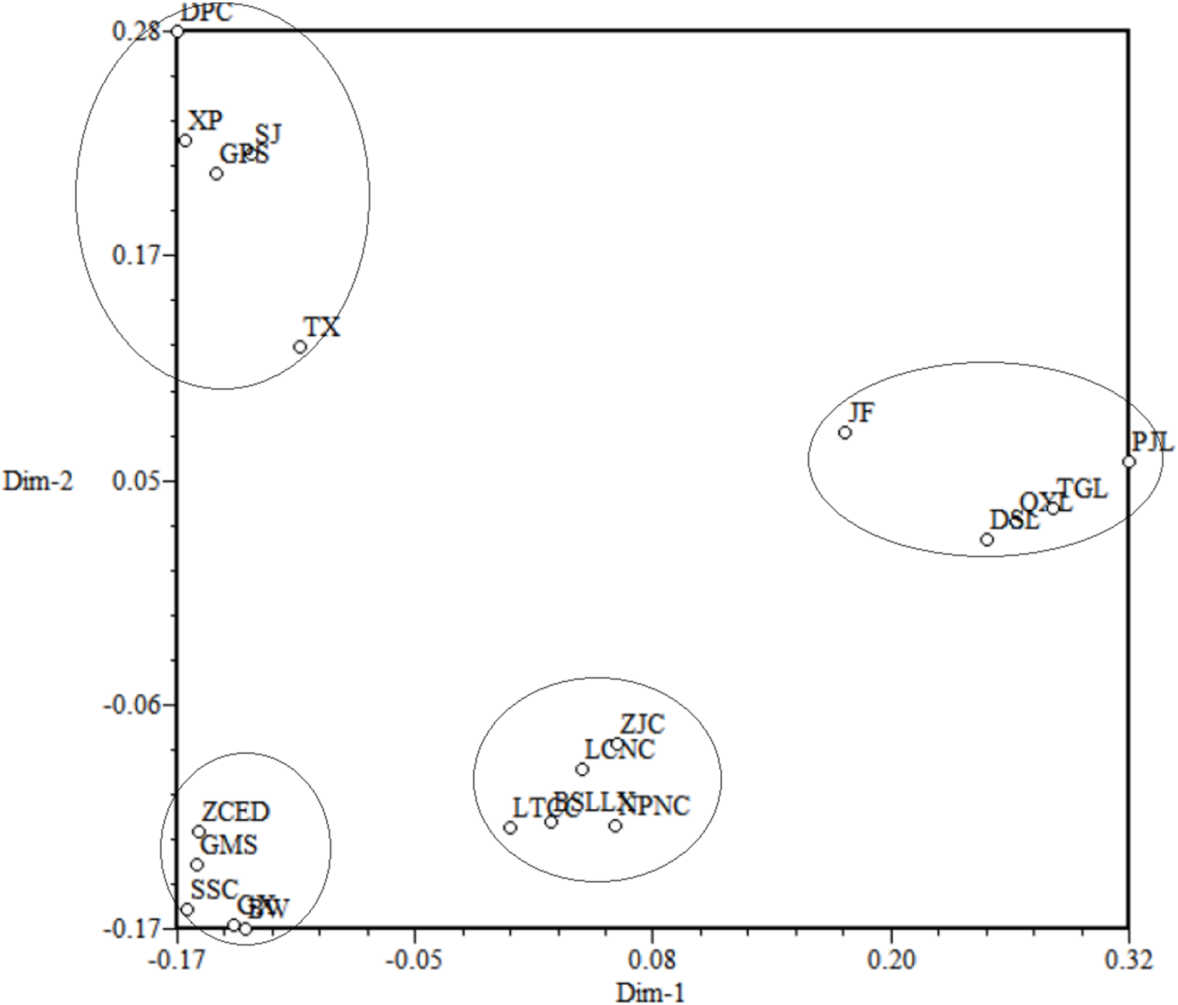
Principal coordinates analysis (PCoA) for 20 populations of *M. oblongifolius*.

Principal Coordinate Analysis was used to construct 3D eigenvectors using the DCENTER module of the NTSYS-pc program to add complementary information to the cluster analysis (Fig. 7). The PCoA for 20 populations of *M. oblongifolius* revealed that these populations were divided into four groups. The results of PCoA were the same from the other cluster analyses as shown above.

**Figure 8:**
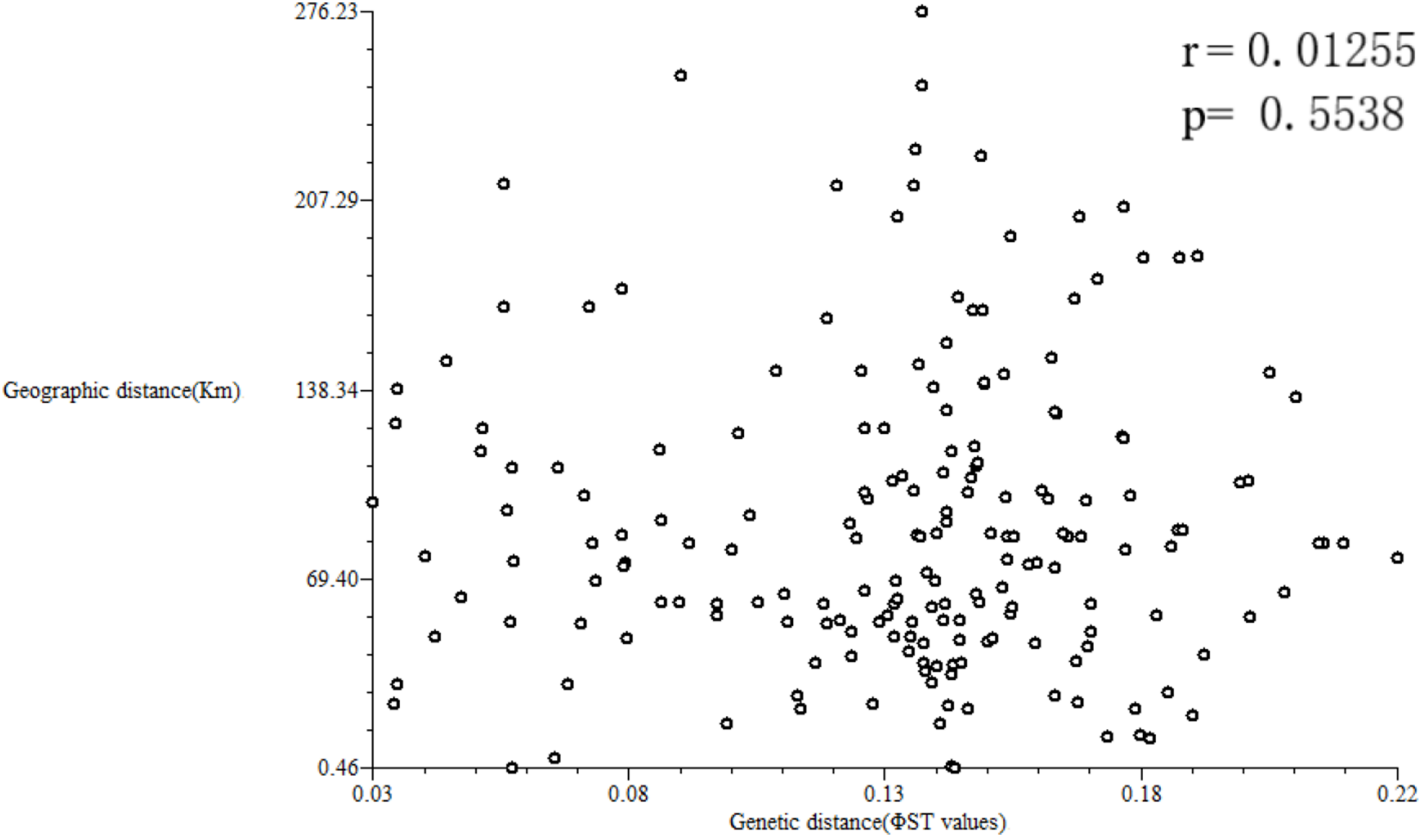
Correlation analysis between geographical and genetic distances.

Geographic distances of the 10 studied populations ranged from 0.456 to 276.232 km, with a mean of 81.63 km (Table S6). A Mantel test was performed to estimate a correlation between the matrices of Nei’s (1978) GDs and of geographical distances using NTSYS-pc version 2.1 (Fig. 8). The results showed a low correlation between geographical and GDs (*r* = 0.01255, *p* = 0.5538).

## Discussion

### Genetic diversity of *M. obongifolius*

From the perspective of modern genetics, species with higher genetic diversity will have a wider natural distribution and stronger environmental adaptability, survivability and evolutionary potential. Genetic variation is a necessary condition for a species to adapt to environmental changes (Liu et al., 2015). Therefore, evaluation of genetic diversity is an essential component in germplasm characterization and collection. In the present study based on SRAP markers, ne, h and I were calculated to be 1.5702, 0.336 and 0.5057 at the species level, respectively. These results were higher than those obtained using ISSR markers (ne = 1.5186, *h* = 0.3068 and *I* = 0.4657). However, SRAP markers produced a lower PPB (83.24%) than ISSR markers (87.94%). Sequence-related amplified polymorphism markers amplify the ORF of plant genome, whereas ISSR markers amplify the repetitive regions. Thus, differences exist in the polymorphism of the two amplification targets (Song et al., 2010). Our results show that both SRAP and ISSR markers can effectively reveal the polymorphism among materials. Mantel’s test showed that the analysis results of SRAP and ISSR were highly and significantly correlated at the population level (*r* = 0.7804, *p* = 0.01).

When compared with other species belonging to the Euphorbiaceae family such as *Jatropha curcas* L. (85.19% for ISSR) (Mavuso et al., 2016) and *Ricinus communis* L. (68.1% for ISSR) (Gajera et al., 2010), our study reveals a relatively high level of PPB (83.24% for SRAP, 87.94% for ISSR) in *M. oblongifolius*. Furthermore, the genetic diversity of *M. oblongifolius* (*h* = 0.336, *I* = 0.5057, SRAP markers and *h* = 0.3068, *I* = 0.4657, ISSR markers) is higher than the average genetic diversity of multiple plant populations detected based on dominant markers such as RAPD, RFLP and ISSR (*h* = 0.22) in the review of Nybom (2004). Genetic diversity also differed somewhat within the 20 populations in this study. Both SRAP and ISSR markers indicated that population TX had the highest genetic diversity and PJL had the lowest. This variation may be due to human activity, random genetic drift and/or inbreeding variation. During our field investigation, we found that population PJL, with the lowest genetic diversity, was a highly damaged population potentially beyond recovery.

### Genetic structure of *M. oblongifolius* populations

Genetic differentiation is an important index of genetic structure. Wright (1951) has previously shown that Gst can be used to determine the degree of genetic differentiation between populations with isoenzymes. In the present study, Gst = 0.2764 for SRAP and Gst = 0.2709 for ISSR were revealed for the 20 populations of *M. oblongifolius*, which were equivalent to or less than the mean values in the Nybom (2004) review of 31 RAPD and six ISSR studies (mean Gst = 0.27 for RAPD, Gst = 0.34 for ISSR), indicating weak genetic differentiation among the populations of *M. oblongifolius*.

Hamrick & Godt (1990) believed that the mating system was the biggest factor affecting plant genetic diversity and genetic differentiation. Among the genetic variation in selfing species, 51% exist across populations (Gst = 0.510), which amounts to five times more than that of wind-borne plants (Gst = 0.099). Nearly 27% of the total genetic variation in the 20 populations of *M. oblongifolius* occurred among populations (Gst = 0.2764 for SRAP, Gst = 0.2709 for ISSR) with weak genetic differentiation among populations. By reviewing Gst values of plant species (based on RAPD data), Nybom (2004) reported an average Gst of 0.22 for 31 outbreeding species and 0.59 for six inbreeding species, and Bussell (1999) reported an average Gst of 0.19 for 29 outbreeding species and 0.63 for six inbreeding species. We supposed that the mating system of *M. oblongifolius* was the outbreeding or the mixing of mating taxa with dominated outbreeding, which may have an influence on extant among-population genetic structure of *M. oblongifolius*.

Gene flow is the movement of genes within and between populations. The intensity of gene flow has an important impact on population differentiation (Grant, 1991). It is generally believed that when Nm > 1, gene flow can prevent genetic differentiation between populations caused by genetic drift. When Nm < 1, genetic drift is the main reason for genetic structure differentiation among populations. The gene flows (Nm) among the *M. oblongifolius* populations were 1.3092 (SRAP) and 1.346 (ISSR), which indicates that the gene exchange was high between populations. Such an exchange could prevent genetic differentiation caused by genetic drift between populations, and may play important roles in the genetic structure of *M. oblongifolius* populations.

If gene flow by mating system and seed dispersal are the main causes of population variation, the closer the geographical distance between two populations is, the smaller the genetic differentiation should be. However, the result of the Mantel test suggested that the distribution of genetic diversity among populations might not be explained by the pronounced geographical distances (*r* = 0.01255, *p* = 0.5538). The populations in this study were clustered according to the degree of similarity of their habitats and had nothing to do with their geographical location, and the populations with similar habitats first clustered together. The pattern of genetic subdivision can be clearly demonstrated in the UPGMA and NJ cluster analysis (see Figs. 4 and 5), in which the populations of *M. oblongifolius* were divided into two groups according to their habitat type (stream banks, darker damper mountain bases and mountainsides). The results of PCoA and Bayesian analysis also support this habitat-specific genetic clustering model (see Figs. 6 and 7).

Local adaptive divergence will occur when the gene flow is weakened, and populations in different habitats experience divergent patterns of selection (Schluter, 2000). Due to their special medicinal significance, wild populations of *M. oblongifolius* have long been subjected to illegal and unrestrained collection, and the number and size of populations have dropped dramatically. This has led to habitat fragmentation and specificity, which weaken the natural gene flow between the populations and promote the adaptation to special habitats to maintain high genetic diversity.

Our evaluation of genetic diversity is an essential step toward the germplasm characterization of *M. oblongifolius*, which will in turn enable better collection and management practices to preserve its diversity.

## Supplemental Information

10.7717/peerj.7173/supp-1 Supplemental Information 1 Sequence and polymorphism of SRAP.

10.7717/peerj.7173/supp-2 Supplemental Information 2 Sequence and polymorphism of ISSR.

10.7717/peerj.7173/supp-3 Supplemental Information 3 Nei’s genetic identity (above diagonal) and genetic distance (below diagonal) on SRAP among 20 population materials of *M. obongifolius* (Nei’s 1978).

10.7717/peerj.7173/supp-4 Supplemental Information 4 Nei’s genetic identity (above diagonal) and genetic distance (below diagonal) on ISSR among 20 population materials of *M. obongifolius* (Nei’s 1978).

10.7717/peerj.7173/supp-5 Supplemental Information 5 Nei’s genetic identity (above diagonal) and genetic distance (below diagonal) combined SRAP and ISSR data among 20 population materials of *M. obongifolius* (Nei’s 1978).

10.7717/peerj.7173/supp-6 Supplemental Information 6 Geographic distance (km) between 20 *M. obongifolius* populations in Hainan Island, China.

## Additional Information and Declarations

### Data Availability

The following information was supplied regarding data availability: The raw data are available in Tables 1, 2, 3; Table S6.
