# Supplementary material for: Analysis of genetic population structure and diversity in Mallotus oblongifolius using ISSR and SRAP markers
Source: PeerJ. 2019 Jun 21;7:e7173. doi: 10.7717/peerj.7173 (PMC6590392; doi:10.7717/peerj.7173)
Supplement: Supplemental Information 1 [file peerj-07-7173-s001.docx]

| Primers | Sequence(5' to 3') | Annealing temperature | Bands | Polymorphic bands | Percentage Polymorphic bands (%) |
| --- | --- | --- | --- | --- | --- |
| ME1-EM1 | TGAGTCCAAACCGGATA | 48℃ | 15 | 15 | 100 |
|  | GACTGCGTACGAATTAAT |  |  |  |  |
| ME1-EM2 | TGAGTCCAAACCGGATA | 48℃ | 13 | 10 | 76.92 |
|  | GACTGCGTACGAATTTGC |  |  |  |  |
| ME1-EM3 | TGAGTCCAAACCGGATA | 48℃ | 16 | 13 | 81.25 |
|  | GACTGCGTACGAATTGAC |  |  |  |  |
| ME1-EM4 | TGAGTCCAAACCGGATA | 48℃ | 17 | 16 | 94.12 |
|  | GACTGCGTACGAATTACG |  |  |  |  |
| ME1-EM9 | TGAGTCCAAACCGGATA | 48℃ | 20 | 19 | 95 |
|  | GACTGCGTACGAATTCGA |  |  |  |  |
| ME1-EM10 | TGAGTCCAAACCGGATA | 48℃ | 17 | 14 | 82.35 |
|  | GACTGCGTACGAATTCAG |  |  |  |  |
| ME2-EM9 | TGAGTCCAAACCGGAGC | 48℃ | 9 | 3 | 33.33 |
|  | GACTGCGTACGAATTCGA |  |  |  |  |
| ME5-EM8 | TGAGTCCAAACCGGAAG | 48℃ | 14 | 11 | 78.57 |
|  | GACTGCGTACGAATTCTG |  |  |  |  |
| ME6-EM5 | TGAGTCCAAACCGGTAA | 48℃ | 12 | 11 | 91.67 |
|  | GACTGCGTACGAATTAAC |  |  |  |  |
| ME7-EM1 | TGAGTCCAAACCGGTCC | 48℃ | 10 | 9 | 90 |
|  | GACTGCGTACGAATTAAT |  |  |  |  |
| ME7-EM2 | TGAGTCCAAACCGGTCC | 48℃ | 10 | 6 | 60 |
|  | GACTGCGTACGAATTTGC |  |  |  |  |
| ME5-EM2 | TGAGTCCAAACCGGAAG | 48℃ | 15 | 13 | 86.67 |
|  | GACTGCGTACGAATTTGC |  |  |  |  |
| ME4-EM2 | TGAGTCCAAACCGGAAA | 48℃ | 15 | 13 | 86.67 |
|  | GACTGCGTACGAATTTGC |  |  |  |  |
| ME9-EM1 | TGAGTCCAAACCGGACG | 48℃ | 14 | 11 | 78.57 |
|  | GACTGCGTACGAATTAAT |  |  |  |  |
| Mean | | | 14.1 | 11.7 | 82.98 |
| Total | | | 197 | 164 | 83.24 |
