# Supplementary material for: Analysis of genetic population structure and diversity in Mallotus oblongifolius using ISSR and SRAP markers
Source: PeerJ. 2019 Jun 21;7:e7173. doi: 10.7717/peerj.7173 (PMC6590392; doi:10.7717/peerj.7173)
Supplement: Supplemental Information 2 [file peerj-07-7173-s002.docx]

| Primers | Sequence | Annealing temperature | Bands | Polymorphic bands | Percentage Polymorphic bands (%) |
| --- | --- | --- | --- | --- | --- |
|  | (5' to 3') |  |  |  |  |
| ISSR-02 | GTGCGTGCGTGCGTGC | 45℃ | 10 | 8 | 80 |
| ISSR-03 | GACAGACAGACAGACAA | 48℃ | 13 | 11 | 84.61 |
| UBC-812 | GAGAGAGAGAGAGAGAA | 48℃ | 14 | 12 | 85.71 |
| UBC-818 | CACACACACACACACAG | 48℃ | 11 | 10 | 90.91 |
| UBC-826 | ACACACACACACACACG | 48℃ | 13 | 11 | 84.61 |
| UBC-835 | AGAGAGAGAGAGAGAGYC | 51℃ | 13 | 10 | 76.92 |
| UBC-840 | GAGAGAGAGAGAGAGAYT | 51℃ | 17 | 17 | 100 |
| UBC-841 | GAGAGAGAGAGAGAGAYC | 51℃ | 17 | 13 | 76.47 |
| UBC-855 | ACACACACACACACACYT | 51℃ | 17 | 17 | 100 |
| UBC-891 | HVHTGTGTGTGTGTGTG | 52℃ | 16 | 15 | 93.75 |
| Mean | | | 14.1 | 12.4 | 87.94 |
| Total | | | 141 | 124 | 87.94 |
