# Supplementary material for: Analysis of genetic population structure and diversity in Mallotus oblongifolius using ISSR and SRAP markers
Source: PeerJ. 2019 Jun 21;7:e7173. doi: 10.7717/peerj.7173 (PMC6590392; doi:10.7717/peerj.7173)
Supplement: Supplemental Information 3 [file peerj-07-7173-s003.docx]

| Materials | GPS | XP | DPC | SJ | TX | ZCED | GMS | SSC | GX | BW | ZJC | LCNC | BSLLX | LTCC | NPNC | TGL | JF | DSL | QXL | PJL |
| --- | --- | --- | --- | --- | --- | --- | --- | --- | --- | --- | --- | --- | --- | --- | --- | --- | --- | --- | --- | --- |
| GPS | 1.0000 | 0.9563 | 0.9326 | 0.9362 | 0.9205 | 0.8713 | 0.865 | 0.8556 | 0.8505 | 0.8538 | 0.8586 | 0.8752 | 0.8489 | 0.8629 | 0.8414 | 0.8134 | 0.8414 | 0.8375 | 0.8262 | 0.7976 |
| XP | 0.0447 | 1.0000 | 0.9751 | 0.9674 | 0.95 | 0.8711 | 0.8832 | 0.8653 | 0.8581 | 0.8634 | 0.8692 | 0.8802 | 0.8676 | 0.8736 | 0.8706 | 0.8175 | 0.8645 | 0.8563 | 0.8475 | 0.8138 |
| DPC | 0.0698 | 0.0252 | 1.0000 | 0.963 | 0.9308 | 0.8582 | 0.8634 | 0.842 | 0.8397 | 0.8332 | 0.8356 | 0.8564 | 0.8536 | 0.8541 | 0.843 | 0.8042 | 0.8385 | 0.8299 | 0.8235 | 0.7825 |
| SJ | 0.0659 | 0.0332 | 0.0377 | 1.0000 | 0.9731 | 0.8813 | 0.8705 | 0.8757 | 0.8633 | 0.8589 | 0.8741 | 0.88 | 0.8702 | 0.874 | 0.8777 | 0.8142 | 0.8519 | 0.8561 | 0.8501 | 0.8157 |
| TX | 0.0828 | 0.0513 | 0.0717 | 0.0272 | 1.0000 | 0.9053 | 0.9035 | 0.9053 | 0.8934 | 0.8919 | 0.8887 | 0.9004 | 0.8826 | 0.8929 | 0.9054 | 0.8595 | 0.8901 | 0.8966 | 0.8801 | 0.8533 |
| ZCED | 0.1378 | 0.138 | 0.1529 | 0.1263 | 0.0995 | 1.0000 | 0.9471 | 0.9449 | 0.9347 | 0.9217 | 0.8722 | 0.9041 | 0.8937 | 0.8911 | 0.8846 | 0.8413 | 0.8678 | 0.882 | 0.8567 | 0.8324 |
| GMS | 0.145 | 0.1242 | 0.1469 | 0.1387 | 0.1015 | 0.0544 | 1.0000 | 0.9446 | 0.9418 | 0.9366 | 0.8747 | 0.8922 | 0.8805 | 0.8946 | 0.897 | 0.8373 | 0.8681 | 0.8745 | 0.8496 | 0.814 |
| SSC | 0.156 | 0.1447 | 0.1719 | 0.1328 | 0.0995 | 0.0566 | 0.057 | 1.0000 | 0.9722 | 0.9561 | 0.8769 | 0.9066 | 0.8971 | 0.8952 | 0.8843 | 0.8306 | 0.8566 | 0.8673 | 0.857 | 0.8316 |
| GX | 0.162 | 0.1531 | 0.1747 | 0.147 | 0.1128 | 0.0675 | 0.06 | 0.0282 | 1.0000 | 0.969 | 0.8785 | 0.8971 | 0.8902 | 0.8972 | 0.8808 | 0.8415 | 0.8597 | 0.8718 | 0.8665 | 0.8275 |
| BW | 0.158 | 0.1469 | 0.1824 | 0.1521 | 0.1144 | 0.0816 | 0.0655 | 0.0449 | 0.0315 | 1.0000 | 0.8626 | 0.8896 | 0.8744 | 0.8838 | 0.8747 | 0.8365 | 0.8689 | 0.869 | 0.8574 | 0.8114 |
| ZJC | 0.1524 | 0.1402 | 0.1796 | 0.1345 | 0.118 | 0.1367 | 0.1339 | 0.1313 | 0.1296 | 0.1479 | 1.0000 | 0.9442 | 0.9122 | 0.907 | 0.9306 | 0.8502 | 0.8582 | 0.8775 | 0.8533 | 0.8322 |
| LCNC | 0.1333 | 0.1277 | 0.155 | 0.1278 | 0.105 | 0.1008 | 0.1141 | 0.098 | 0.1086 | 0.117 | 0.0574 | 1.0000 | 0.9722 | 0.9577 | 0.9191 | 0.862 | 0.8811 | 0.8831 | 0.858 | 0.843 |
| BSLLX | 0.1639 | 0.142 | 0.1583 | 0.1391 | 0.1249 | 0.1124 | 0.1273 | 0.1086 | 0.1163 | 0.1342 | 0.0919 | 0.0282 | 1.0000 | 0.9617 | 0.9192 | 0.8441 | 0.8589 | 0.8699 | 0.8413 | 0.8335 |
| LTCC | 0.1475 | 0.1352 | 0.1577 | 0.1347 | 0.1133 | 0.1153 | 0.1113 | 0.1107 | 0.1084 | 0.1235 | 0.0976 | 0.0432 | 0.039 | 1.0000 | 0.9257 | 0.851 | 0.8742 | 0.8691 | 0.8574 | 0.8302 |
| NPNC | 0.1727 | 0.1385 | 0.1708 | 0.1305 | 0.0994 | 0.1226 | 0.1087 | 0.123 | 0.1269 | 0.1338 | 0.0719 | 0.0844 | 0.0842 | 0.0772 | 1.0000 | 0.8665 | 0.8796 | 0.8946 | 0.8614 | 0.8554 |
| TGL | 0.2066 | 0.2015 | 0.218 | 0.2055 | 0.1514 | 0.1728 | 0.1776 | 0.1856 | 0.1725 | 0.1785 | 0.1623 | 0.1485 | 0.1695 | 0.1614 | 0.1433 | 1.0000 | 0.9232 | 0.9618 | 0.9276 | 0.9296 |
| JF | 0.1727 | 0.1456 | 0.1762 | 0.1603 | 0.1164 | 0.1418 | 0.1415 | 0.1548 | 0.1512 | 0.1405 | 0.1529 | 0.1265 | 0.1521 | 0.1345 | 0.1283 | 0.0799 | 1.0000 | 0.944 | 0.9206 | 0.9041 |
| DSL | 0.1773 | 0.1551 | 0.1864 | 0.1553 | 0.1092 | 0.1255 | 0.1341 | 0.1424 | 0.1372 | 0.1404 | 0.1306 | 0.1244 | 0.1394 | 0.1403 | 0.1113 | 0.0389 | 0.0576 | 1.0000 | 0.9659 | 0.9417 |
| QXL | 0.1909 | 0.1655 | 0.1942 | 0.1623 | 0.1277 | 0.1546 | 0.163 | 0.1543 | 0.1433 | 0.1539 | 0.1586 | 0.1531 | 0.1728 | 0.1539 | 0.1492 | 0.0752 | 0.0828 | 0.0347 | 1.0000 | 0.9312 |
| PJL | 0.2261 | 0.2061 | 0.2453 | 0.2038 | 0.1586 | 0.1834 | 0.2057 | 0.1844 | 0.1894 | 0.209 | 0.1837 | 0.1708 | 0.1821 | 0.186 | 0.1562 | 0.073 | 0.1008 | 0.06 | 0.0713 | 1.0000 |
