# Supplementary material for: Analysis of genetic population structure and diversity in Mallotus oblongifolius using ISSR and SRAP markers
Source: PeerJ. 2019 Jun 21;7:e7173. doi: 10.7717/peerj.7173 (PMC6590392; doi:10.7717/peerj.7173)
Supplement: Supplemental Information 4 [file peerj-07-7173-s004.docx]

| Materials | GPS | XP | DPC | SJ | TX | ZCED | GMS | SSC | GX | BW | ZJC | LCNC | BSLLX | LTCC | NPNC | TGL | JF | DSL | QXL | PJL |
| --- | --- | --- | --- | --- | --- | --- | --- | --- | --- | --- | --- | --- | --- | --- | --- | --- | --- | --- | --- | --- |
| GPS | 1.0000 | 0.9515 | 0.9481 | 0.9407 | 0.9344 | 0.8829 | 0.8921 | 0.8959 | 0.9097 | 0.89 | 0.889 | 0.8779 | 0.8794 | 0.8835 | 0.8564 | 0.8688 | 0.9047 | 0.8874 | 0.875 | 0.8433 |
| XP | 0.0497 | 1.0000 | 0.9723 | 0.9629 | 0.9614 | 0.8986 | 0.9148 | 0.9157 | 0.924 | 0.9135 | 0.8807 | 0.8846 | 0.8792 | 0.8823 | 0.8648 | 0.8747 | 0.9067 | 0.8852 | 0.8697 | 0.8486 |
| DPC | 0.0533 | 0.0281 | 1.0000 | 0.9606 | 0.9356 | 0.8627 | 0.8735 | 0.883 | 0.8931 | 0.8698 | 0.8547 | 0.8628 | 0.8547 | 0.8575 | 0.8374 | 0.8617 | 0.8901 | 0.8729 | 0.8619 | 0.8437 |
| SJ | 0.0612 | 0.0378 | 0.0402 | 1.0000 | 0.9769 | 0.877 | 0.9092 | 0.9045 | 0.9096 | 0.9058 | 0.8909 | 0.8928 | 0.8924 | 0.891 | 0.8758 | 0.8981 | 0.9261 | 0.9081 | 0.8927 | 0.8758 |
| TX | 0.0679 | 0.0393 | 0.0666 | 0.0234 | 1.0000 | 0.9138 | 0.95 | 0.9253 | 0.9339 | 0.9446 | 0.9009 | 0.8779 | 0.8908 | 0.9029 | 0.8942 | 0.8865 | 0.9288 | 0.9107 | 0.8773 | 0.8667 |
| ZCED | 0.1245 | 0.107 | 0.1476 | 0.1313 | 0.0902 | 1.0000 | 0.9581 | 0.9406 | 0.9447 | 0.9479 | 0.85 | 0.8346 | 0.8452 | 0.8566 | 0.8596 | 0.8348 | 0.8376 | 0.8522 | 0.8277 | 0.8014 |
| GMS | 0.1142 | 0.0891 | 0.1353 | 0.0952 | 0.0513 | 0.0428 | 1.0000 | 0.9628 | 0.967 | 0.9769 | 0.8912 | 0.867 | 0.8857 | 0.9018 | 0.91 | 0.8555 | 0.8645 | 0.8852 | 0.855 | 0.8289 |
| SSC | 0.1099 | 0.088 | 0.1245 | 0.1004 | 0.0777 | 0.0612 | 0.0379 | 1.0000 | 0.9832 | 0.9622 | 0.8668 | 0.8712 | 0.8789 | 0.8889 | 0.8693 | 0.8533 | 0.8561 | 0.8689 | 0.8527 | 0.8178 |
| GX | 0.0947 | 0.0791 | 0.1131 | 0.0947 | 0.0683 | 0.0568 | 0.0336 | 0.0169 | 1.0000 | 0.9807 | 0.8896 | 0.8818 | 0.8904 | 0.9109 | 0.8927 | 0.8657 | 0.8692 | 0.8852 | 0.8745 | 0.839 |
| BW | 0.1165 | 0.0905 | 0.1395 | 0.0989 | 0.057 | 0.0535 | 0.0234 | 0.0386 | 0.0195 | 1.0000 | 0.9106 | 0.8718 | 0.8835 | 0.9058 | 0.9226 | 0.8668 | 0.867 | 0.8968 | 0.867 | 0.8436 |
| ZJC | 0.1177 | 0.1271 | 0.157 | 0.1155 | 0.1044 | 0.1626 | 0.1152 | 0.1429 | 0.117 | 0.0936 | 1.0000 | 0.9307 | 0.9221 | 0.9241 | 0.9591 | 0.8992 | 0.8926 | 0.9191 | 0.9091 | 0.8883 |
| LCNC | 0.1302 | 0.1226 | 0.1476 | 0.1134 | 0.1302 | 0.1808 | 0.1427 | 0.1379 | 0.1258 | 0.1372 | 0.0719 | 1.0000 | 0.973 | 0.9451 | 0.9138 | 0.8837 | 0.8984 | 0.901 | 0.9241 | 0.8622 |
| BSLLX | 0.1285 | 0.1288 | 0.157 | 0.1138 | 0.1157 | 0.1682 | 0.1213 | 0.1291 | 0.116 | 0.1239 | 0.0811 | 0.0274 | 1.0000 | 0.9699 | 0.9245 | 0.8742 | 0.8975 | 0.9016 | 0.9269 | 0.844 |
| LTCC | 0.1239 | 0.1252 | 0.1538 | 0.1154 | 0.1022 | 0.1547 | 0.1033 | 0.1178 | 0.0933 | 0.0989 | 0.0789 | 0.0564 | 0.0306 | 1.0000 | 0.9408 | 0.8691 | 0.9051 | 0.8928 | 0.9187 | 0.8313 |
| NPNC | 0.155 | 0.1453 | 0.1775 | 0.1326 | 0.1118 | 0.1513 | 0.0943 | 0.14 | 0.1135 | 0.0805 | 0.0417 | 0.0902 | 0.0785 | 0.061 | 1.0000 | 0.8783 | 0.8672 | 0.9192 | 0.9037 | 0.8592 |
| TGL | 0.1406 | 0.1339 | 0.1489 | 0.1075 | 0.1205 | 0.1806 | 0.156 | 0.1586 | 0.1442 | 0.143 | 0.1063 | 0.1236 | 0.1344 | 0.1403 | 0.1297 | 1.0000 | 0.9203 | 0.9508 | 0.9398 | 0.9274 |
| JF | 0.1001 | 0.098 | 0.1164 | 0.0768 | 0.0738 | 0.1773 | 0.1457 | 0.1553 | 0.1402 | 0.1427 | 0.1136 | 0.1071 | 0.1081 | 0.0998 | 0.1424 | 0.083 | 1.0000 | 0.9315 | 0.9345 | 0.8952 |
| DSL | 0.1195 | 0.122 | 0.136 | 0.0964 | 0.0936 | 0.16 | 0.122 | 0.1405 | 0.122 | 0.1089 | 0.0844 | 0.1042 | 0.1036 | 0.1134 | 0.0842 | 0.0504 | 0.071 | 1.0000 | 0.9726 | 0.9555 |
| QXL | 0.1335 | 0.1396 | 0.1486 | 0.1135 | 0.1309 | 0.1891 | 0.1567 | 0.1594 | 0.1341 | 0.1428 | 0.0953 | 0.0789 | 0.0759 | 0.0848 | 0.1012 | 0.0621 | 0.0677 | 0.0278 | 1.0000 | 0.942 |
| PJL | 0.1704 | 0.1642 | 0.1699 | 0.1326 | 0.1431 | 0.2213 | 0.1877 | 0.2012 | 0.1755 | 0.17 | 0.1185 | 0.1483 | 0.1696 | 0.1847 | 0.1517 | 0.0754 | 0.1107 | 0.0455 | 0.0597 | 1.0000 |
