# Supplementary material for: Analysis of genetic population structure and diversity in Mallotus oblongifolius using ISSR and SRAP markers
Source: PeerJ. 2019 Jun 21;7:e7173. doi: 10.7717/peerj.7173 (PMC6590392; doi:10.7717/peerj.7173)
Supplement: Supplemental Information 5 [file peerj-07-7173-s005.docx]

| Materials | GPS | XP | DPC | SJ | TX | ZCED | GMS | SSC | GX | BW | ZJC | LCNC | BSLLX | LTCC | NPNC | TGL | JF | DSL | QXL | PJL |
| --- | --- | --- | --- | --- | --- | --- | --- | --- | --- | --- | --- | --- | --- | --- | --- | --- | --- | --- | --- | --- |
| GPS | 1.0000 | 0.9460 | 0.9317 | 0.9300 | 0.9185 | 0.8698 | 0.8692 | 0.8657 | 0.8685 | 0.8608 | 0.8640 | 0.8694 | 0.8551 | 0.8652 | 0.8403 | 0.8305 | 0.8608 | 0.8516 | 0.8394 | 0.8080 |
| XP | 0.0555 | 1.0000 | 0.9655 | 0.9563 | 0.9461 | 0.8759 | 0.8887 | 0.8793 | 0.8786 | 0.8757 | 0.8663 | 0.8745 | 0.8654 | 0.8704 | 0.8600 | 0.8350 | 0.8747 | 0.8613 | 0.8491 | 0.8192 |
| DPC | 0.0707 | 0.0351 | 1.0000 | 0.9537 | 0.9252 | 0.8542 | 0.8609 | 0.8530 | 0.8559 | 0.8410 | 0.8370 | 0.8527 | 0.8479 | 0.8497 | 0.8336 | 0.8226 | 0.8536 | 0.8417 | 0.8329 | 0.8000 |
| SJ | 0.0726 | 0.0447 | 0.0474 | 1.0000 | 0.9656 | 0.8725 | 0.8788 | 0.8802 | 0.8754 | 0.8696 | 0.8730 | 0.8777 | 0.8721 | 0.8741 | 0.8684 | 0.8423 | 0.8751 | 0.8703 | 0.8599 | 0.8311 |
| TX | 0.0850 | 0.0554 | 0.0778 | 0.0350 | 1.0000 | 0.9020 | 0.9153 | 0.9063 | 0.9033 | 0.9052 | 0.8861 | 0.8835 | 0.8791 | 0.8903 | 0.8924 | 0.8643 | 0.8988 | 0.8952 | 0.8711 | 0.8494 |
| ZCED | 0.1395 | 0.1325 | 0.1576 | 0.1364 | 0.1031 | 1.0000 | 0.9447 | 0.9364 | 0.9325 | 0.9248 | 0.8562 | 0.8681 | 0.8670 | 0.8708 | 0.8670 | 0.8333 | 0.8487 | 0.8633 | 0.8379 | 0.8111 |
| GMS | 0.1402 | 0.1179 | 0.1498 | 0.1292 | 0.0885 | 0.0569 | 1.0000 | 0.9447 | 0.9450 | 0.9444 | 0.8744 | 0.8744 | 0.8759 | 0.8910 | 0.8946 | 0.8390 | 0.8595 | 0.8722 | 0.8447 | 0.8116 |
| SSC | 0.1443 | 0.1286 | 0.1590 | 0.1276 | 0.0983 | 0.0657 | 0.0569 | 1.0000 | 0.9694 | 0.9497 | 0.8656 | 0.8846 | 0.8828 | 0.8862 | 0.8705 | 0.8345 | 0.8499 | 0.8615 | 0.8482 | 0.8171 |
| GX | 0.1410 | 0.1294 | 0.1556 | 0.1331 | 0.1017 | 0.0699 | 0.0566 | 0.0310 | 1.0000 | 0.9650 | 0.8763 | 0.8840 | 0.8840 | 0.8968 | 0.8786 | 0.8461 | 0.8573 | 0.8711 | 0.8629 | 0.8239 |
| BW | 0.1499 | 0.1327 | 0.1731 | 0.1397 | 0.0996 | 0.0782 | 0.0572 | 0.0516 | 0.0356 | 1.0000 | 0.8748 | 0.8738 | 0.8704 | 0.8854 | 0.8864 | 0.8421 | 0.8600 | 0.8728 | 0.8530 | 0.8153 |
| ZJC | 0.1462 | 0.1436 | 0.1780 | 0.1358 | 0.1209 | 0.1552 | 0.1342 | 0.1443 | 0.1321 | 0.1338 | 1.0000 | 0.9303 | 0.9090 | 0.9071 | 0.9346 | 0.8645 | 0.8659 | 0.8882 | 0.8698 | 0.8471 |
| LCNC | 0.1400 | 0.1341 | 0.1594 | 0.1304 | 0.1239 | 0.1415 | 0.1343 | 0.1226 | 0.1233 | 0.1349 | 0.0722 | 1.0000 | 0.9652 | 0.9455 | 0.9089 | 0.8650 | 0.8815 | 0.8840 | 0.8790 | 0.8420 |
| BSLLX | 0.1566 | 0.1446 | 0.1650 | 0.1368 | 0.1289 | 0.1427 | 0.1324 | 0.1247 | 0.1233 | 0.1388 | 0.0954 | 0.0355 | 1.0000 | 0.9582 | 0.9136 | 0.8510 | 0.8687 | 0.8768 | 0.8706 | 0.8293 |
| LTCC | 0.1448 | 0.1388 | 0.1629 | 0.1346 | 0.1162 | 0.1383 | 0.1154 | 0.1208 | 0.1089 | 0.1218 | 0.0975 | 0.0561 | 0.0427 | 1.0000 | 0.9244 | 0.8530 | 0.8807 | 0.8729 | 0.8764 | 0.8224 |
| NPNC | 0.1740 | 0.1508 | 0.1820 | 0.1411 | 0.1139 | 0.1427 | 0.1113 | 0.1387 | 0.1294 | 0.1206 | 0.0676 | 0.0955 | 0.0903 | 0.0786 | 1.0000 | 0.8646 | 0.8666 | 0.8975 | 0.8717 | 0.8473 |
| TGL | 0.1858 | 0.1803 | 0.1953 | 0.1716 | 0.1458 | 0.1824 | 0.1756 | 0.1809 | 0.1671 | 0.1719 | 0.1456 | 0.1451 | 0.1613 | 0.1589 | 0.1455 | 1.0000 | 0.9150 | 0.9501 | 0.9252 | 0.9189 |
| JF | 0.1499 | 0.1339 | 0.1583 | 0.1334 | 0.1067 | 0.1640 | 0.1514 | 0.1627 | 0.1540 | 0.1508 | 0.1440 | 0.1261 | 0.1408 | 0.1271 | 0.1432 | 0.0888 | 1.0000 | 0.9310 | 0.9184 | 0.8903 |
| DSL | 0.1606 | 0.1493 | 0.1723 | 0.1389 | 0.1107 | 0.1470 | 0.1368 | 0.1490 | 0.1380 | 0.1361 | 0.1186 | 0.1233 | 0.1315 | 0.1360 | 0.1081 | 0.0512 | 0.0715 | 1.0000 | 0.9602 | 0.9372 |
| QXL | 0.1751 | 0.1635 | 0.1829 | 0.1509 | 0.1380 | 0.1769 | 0.1688 | 0.1647 | 0.1474 | 0.1590 | 0.1395 | 0.1289 | 0.1385 | 0.1320 | 0.1374 | 0.0778 | 0.0852 | 0.0406 | 1.0000 | 0.9249 |
| PJL | 0.2132 | 0.1994 | 0.2232 | 0.1849 | 0.1632 | 0.2094 | 0.2087 | 0.2020 | 0.1937 | 0.2043 | 0.1659 | 0.1719 | 0.1871 | 0.1956 | 0.1658 | 0.0846 | 0.1162 | 0.0649 | 0.0781 | 1.0000 |
