# Supplementary material for: Analysis of genetic population structure and diversity in Mallotus oblongifolius using ISSR and SRAP markers
Source: PeerJ. 2019 Jun 21;7:e7173. doi: 10.7717/peerj.7173 (PMC6590392; doi:10.7717/peerj.7173)
Supplement: Supplemental Information 6 [file peerj-07-7173-s006.docx]

| Materials | GPS | XP | DPC | SJ | TX | ZCED | GMS | SSC | GX | BW | ZJC | LCNC | BSLLX | LTCC | NPNC | TGL | JF | DSL | QXL | PJL |
| --- | --- | --- | --- | --- | --- | --- | --- | --- | --- | --- | --- | --- | --- | --- | --- | --- | --- | --- | --- | --- |
| GPS | 0.000 |  |  |  |  |  |  |  |  |  |  |  |  |  |  |  |  |  |  |  |
| XP | 213.615 | 0.000 |  |  |  |  |  |  |  |  |  |  |  |  |  |  |  |  |  |  |
| DPC | 99.668 | 126.503 | 0.000 |  |  |  |  |  |  |  |  |  |  |  |  |  |  |  |  |  |
| SJ | 68.978 | 148.909 | 62.677 | 0.000 |  |  |  |  |  |  |  |  |  |  |  |  |  |  |  |  |
| TX | 61.147 | 168.762 | 85.583 | 24.012 | 0.000 |  |  |  |  |  |  |  |  |  |  |  |  |  |  |  |
| ZCED | 1.052 | 212.954 | 98.752 | 68.586 | 61.117 | 0.000 |  |  |  |  |  |  |  |  |  |  |  |  |  |  |
| GMS | 0.648 | 213.094 | 99.042 | 68.592 | 60.964 | 0.456 | 0.000 |  |  |  |  |  |  |  |  |  |  |  |  |  |
| SSC | 110.663 | 105.284 | 27.005 | 56.279 | 80.226 | 109.892 | 110.092 | 0.000 |  |  |  |  |  |  |  |  |  |  |  |  |
| GX | 54.419 | 201.415 | 75.174 | 85.278 | 92.814 | 53.424 | 53.879 | 97.352 | 0.000 |  |  |  |  |  |  |  |  |  |  |  |
| BW | 76.392 | 226.093 | 99.934 | 116.309 | 122.901 | 75.570 | 76.004 | 124.445 | 31.041 | 0.000 |  |  |  |  |  |  |  |  |  |  |
| ZJC | 46.671 | 167.255 | 56.240 | 31.512 | 41.546 | 45.934 | 46.114 | 64.061 | 53.944 | 84.924 | 0.000 |  |  |  |  |  |  |  |  |  |
| LCNC | 38.346 | 249.540 | 130.044 | 107.214 | 98.718 | 38.664 | 38.688 | 145.036 | 65.463 | 71.766 | 82.508 | 0.000 |  |  |  |  |  |  |  |  |
| BSLLX | 101.969 | 112.116 | 44.881 | 37.916 | 60.573 | 101.369 | 101.477 | 24.194 | 101.299 | 130.957 | 56.548 | 138.912 | 0.000 |  |  |  |  |  |  |  |
| LTCC | 61.247 | 155.210 | 39.541 | 36.259 | 53.421 | 60.400 | 60.645 | 50.325 | 53.714 | 84.215 | 16.821 | 94.713 | 48.240 | 0.000 |  |  |  |  |  |  |
| NPNC | 22.452 | 194.552 | 87.638 | 47.435 | 38.933 | 22.315 | 22.203 | 94.129 | 62.392 | 89.648 | 31.449 | 60.658 | 82.439 | 48.108 | 0.000 |  |  |  |  |  |
| TGL | 187.165 | 28.287 | 105.054 | 121.344 | 140.715 | 186.546 | 186.664 | 81.403 | 178.686 | 204.926 | 141.211 | 223.711 | 85.238 | 130.168 | 167.548 | 0.000 |  |  |  |  |
| JF | 85.065 | 276.232 | 150.121 | 147.745 | 145.306 | 84.874 | 85.109 | 171.587 | 74.947 | 56.822 | 117.856 | 54.104 | 172.381 | 124.263 | 106.484 | 252.974 | 0.000 |  |  |  |
| DSL | 86.200 | 144.052 | 80.046 | 23.498 | 27.134 | 86.017 | 85.930 | 66.474 | 108.519 | 139.508 | 54.584 | 124.377 | 43.083 | 59.380 | 63.760 | 115.804 | 168.352 | 0.000 |  |  |
| QXL | 12.512 | 201.695 | 87.201 | 59.015 | 54.753 | 11.652 | 11.903 | 98.322 | 47.868 | 73.540 | 34.490 | 48.670 | 90.414 | 48.748 | 16.752 | 175.464 | 90.684 | 78.005 | 0.000 |  |
| PJL | 82.857 | 144.956 | 77.092 | 19.527 | 24.826 | 82.648 | 82.571 | 64.552 | 104.534 | 135.526 | 50.603 | 121.103 | 41.639 | 55.499 | 60.449 | 116.764 | 164.656 | 3.989 | 74.431 | 0.000 |
